# Energy-Protein Supplementation and Lactation Affect Fatty Acid Profile of Liver and Adipose Tissue of Dairy Cows

**DOI:** 10.3390/molecules23030618

**Published:** 2018-03-09

**Authors:** Anna M. Brzozowska, Marek Lukaszewicz, Jolanta M. Oprzadek

**Affiliations:** Institute of Genetics and Animal Breeding of the Polish Academy of Sciences, Postepu Str. 36A, 05-552 Jastrzebiec, Poland; aa.brzoz@gmail.com (A.M.B.); m.lukaszewicz@ighz.pl (M.L.)

**Keywords:** energy-protein supplement, parity, lactation stage, liver, adipose tissue, dairy cattle

## Abstract

This article addresses the hypothesis that lactation stage, parity and energy-protein feed additive affect fatty acid composition of blood, liver and adipose tissue of cows. The experiment was conducted on 24 Polish Holstein-Friesian cows divided into two feeding groups. One group of cows was fed solely a total mixed ration, while the other group was fed a ration with the addition of 2 kg of energy-protein supplement per cow/day. During the experiment, the samples of liver, adipose tissue and blood were taken and their fatty acid compositions were determined. Analysis of variance was applied to fatty acid relative weight percentage to determine the effect of the stage of lactation, parity, and energy-protein supplement on the fatty acid composition of the tissues. Stage of lactation had a significant impact on the content of many fatty acids in all examined tissues. We found that parity had no effect on fatty acid composition of blood, whereas it significantly affected C16:1 *c*9 in liver, and C16:1 *c*9 and C18:0 in adipose tissue. Energy-protein supplement significantly affected the content of most fatty acids in blood (e.g., C18:1 *t*11 and C18:3 n-3) and liver (C18:3 n-3, both isomers of conjugated linolenic acid and n-3 fatty acids derived from fish oil), but it did not affect the profile of the adipose tissue of cows. According to our best knowledge, this is the first study showing the relationship between parity, stage of lactation and the composition of fatty acids in blood, liver and adipose tissue of cows.

## 1. Introduction

In recent years, people have become more knowledgeable about food ingredients that may have beneficial effects on their health and prevention of lifestyle diseases. Due to this rising awareness of the impact of individual fatty acids (FA) on human health and increasing interest in using fat additives in cattle nutrition, knowledge of fat digestion and metabolism in ruminants is of increasing importance.

Research has focused mainly on composing such a feeding ration so that milk with health-promoting properties can be obtained from dairy cows [1]. However, it should be noted that the change in the FA profile of milk, which takes place under modified feeding, has a multilateral impact. For example, consumed fatty acids are incorporated into animal tissues, and play an important role in the modification of biophysical properties, activity and functioning of biological membranes [2,3]. Polyunsaturated fatty acids (PUFA) may also have a positive impact on the functioning of the reproductive system. Numerous studies on cattle have shown that increased fat content in a ration increases the number and size of ovarian follicles, and reduces the time to the first ovulation after calving [4]. It was also observed that supplementation with PUFA increases the amount of precursors for the synthesis of steroid hormones (estradiol, progesterone), prostaglandins (PGF2α), and decreases embryo mortality [5,6]. For this reason, it is crucial to understand changes in the composition of tissues that occur during fat supplementation, together with other traits, which may affect this composition, such as parity and stage of lactation. Thus far, there have been no studies which would address the effect of parity or stage of lactation on the fatty acid profile of cow tissues, in contrast to many studies that investigated the impact of these traits on milk FA profile (reviewed by Samková et al. [7]). Therefore, the objective of this study was to determine the effect of lactation stage and parity on fatty acid profile in blood, liver and adipose tissue of cows, together with the effect of a specially designed energy-protein supplement (EPS) on these tissues.

## 2. Results

### 2.1. The Effect of Stage of Lactation and Parity on Fatty Acid Profiles

#### 2.1.1. Blood

Stage of lactation had a significant impact on the content of C16:0 (*p* < 0.01), C16:1 *c*9 (*p* = 0.04), C18:0 (*p* < 0.01), C18:1 *c*9 (*p* < 0.01), C18:2 n-6 (linoleic, LA; *p* < 0.01), C18:3 n-3 (alpha-linolenic, ALA; *p* < 0.01), C20:3 n-3 (*p* < 0.05), and C20:4 n-6 (*p* < 0.01) in blood. C16:0, C16:1 *c*9, C18:1 *c*9, and C20:4 n-6 reached the highest values at the beginning of lactation (up to 120 days)—15.59%, 0.39%, 12.58% and 1.18%, respectively.

The content of C18:0 gradually increased from 28.59% in the early stage of lactation, through 29.31%, up to 30.26% at the end of lactation.

The contents of C18:2 n-6 and C20:3 n-6 were the lowest at the beginning of lactation and increased in the middle of lactation. The amount of C18:2 n-6 was 28.56%, 31.63% and 30.24% in the early, middle and late stage of lactation, respectively, while the content of C20:3 n-6 in these stages was 3.74%, 4.17% and 4.18%. The content of ALA decreased during the lactation from 3.26% in the early stage to 2.69% in the late stage.

Parity of cows had no effect on any of the fatty acids in blood.

#### 2.1.2. Liver

The content of C14:0 (*p* < 0.01), C16:0 (*p* < 0.01), C16:1 *c*9 (*p* < 0.01), C18:0 (*p* < 0.01), C18:1 *c*9 (*p* < 0.01), C18:2 n-6 (*p* < 0.01), C20:0 (*p* < 0.01), C20:3 n-3 (*p* < 0.01), C20:3 n-6 (*p* < 0.01), C22:1 (*p* < 0.01), C20:5 n-3 (eicosapentaenoic, EPA; *p* = 0.01), and C22:5 n-3 (docosapentaenoic, DPA; *p* < 0.01) in liver depended on the stage of lactation. In the early and middle stage of lactation, the fatty acid profile of liver was characterized by higher content of C14:0 (0.63%), C16:0 (11.64%), C16:1 *c*9 (0.76%) and C18:1 *c*9 (13.70%) than at the end of lactation (0.55%, 9.81%, 0.48% and 11.90%, respectively). The opposite trends (lower amounts at the beginning of lactation vs. higher at the end of lactation) were observed in the case of C18:0 (31.38% vs. 32.97%), C18:2 n-6 (11.70% vs. 12.41%), C20:0 (0.08% vs. 0.11%), C20:3 n-3 (7.42% vs. 8.95%), C20:3 n-6 (0.06% vs. 0.09%), C22:1 (1.91% vs. 2.15%) and C22:5 n-3 (5.16% vs. 5.49%). 

Only the content of C16:1 *c*9 in liver increased with parity: 0.51% in the first and 0.74% in subsequent lactations (*p* = 0.04).

#### 2.1.3. Adipose Tissue

Stage of lactation had a significant impact on the content of C10:0 (*p* < 0.05), C15:0 (*p* < 0.01), C18:2 n-6 (*p* = 0.04), C18:2 *c*9, *t*11 (*p* < 0.01) and C20:0 (*p* < 0.01) in subcutaneous fat. The contents of C10:0 and C18:2 n-6 were lower in the early and middle stage of lactation (0.03% and 1.56%, respectively) than at the end of lactation (0.04% and 1.72%, respectively). In turn, the contents of C15:0, C18:2 *c*9, *t*11 and C20:0 were higher in the early and mid-lactation (0.46%, 0.84% and 0.21%, respectively) than at the end of lactation (0.36%, 0.62% and 0.17% respectively).

We found that parity had a significant effect on the content of C10:0 (*p* = 0.04), C14:1 *c*9 (*p* = 0.03), C15:0 (*p* < 0.01), C16:1 *c*9 (*p* < 0.01) and C18:0 (*p* < 0.01) in subcutaneous adipose tissue. The adipose tissue of primiparous cows was characterized by higher content of C10:0 (0.04%), C15:0 (0.47%) and C18:0 (8.65%) than adipose tissue of multiparous cows (0.03%, 0.38% and 6.22%, respectively). At the same time, the fat of primiparous cows was characterized by lower amounts of C14:1 *c*9 (1.77%) and C16:1 *c*9 (7.52%) than fat of multiparous cows (2.26% and 10.82%, respectively).

### 2.2. The Effect of Energy-Protein Supplementation on Fatty Acid Profiles

#### 2.2.1. Blood

**Figure 1:**
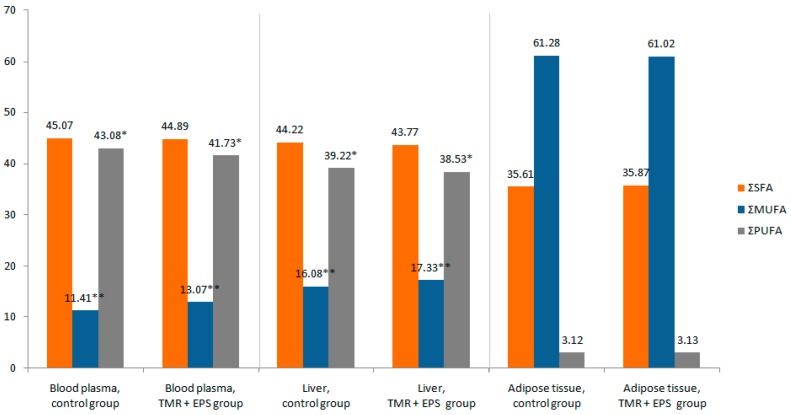
Average weight percentage of the sum of saturated (∑SFA), monounsaturated (∑MUFA) and polyunsaturated (∑PUFA) fatty acids in blood plasma, liver and adipose tissue. Statistically significant differences in the tissue fatty acid composition between the control (total mixed ration) and TMR + EPS (ration plus the energy-protein supplement) groups of cows is indicated by * (*p* < 0.05) and ** (*p* < 0.01).

The energy-protein supplement significantly affected the content of most fatty acids in blood (Figure 1 and Table S2). The highest amounts of C14:0, C15:0, C16:0, C18:2 n-6, and C18:3 n-6 were observed in the control group, whereas the highest amounts of C18:1 *t*11, C18:1 *c*9, C18:3 n-3, C18:2 *c*9, *t*11, C20:3 n-3, C20:4 n-6, C22:1, and C20:5 n-3 were found in the TMR + EPS group. Due to EPS supplementation, the percentage of monounsaturated fatty acids (MUFA) increased at the expense of n-6 PUFA. There was no effect of EPS supplementation on the content of C16:1 *c*9, C18:0, and C22:5 n-3.

#### 2.2.2. Liver

The presence of EPS in the ration influenced the fatty acid content in liver (Figure 1 and Table S2). Higher amounts of C18:1 *t*11, C18:1 *c*9, C18:2 n-6, C18:3 n-3, C18:2 *t*10, *c*12, C18:2 *c*9, *t*11, C20:0, C22:1, C20:5 n-3, C22:5 n-3, and C22:6 n-3 (docosahexaenoic, DHA) were found in the TMR + EPS group than in the control group. In contrast, a lower proportion of C15:0, C20:3 n-3, and C20:4 n-6 was observed in the TMR + EPS group than in the control group. The percentage of PUFA and n-6 decreased, whereas the percentage of MUFA increased during EPS supplementation. The presence of the supplement in the ration had no effect on the contents of the following fatty acids: C14:0, C14:1 *c*9, C16:0, C16:1 *c*9, C18:0, C18:3 n-6, C20:1, and C20:3 n-6.

#### 2.2.3. Adipose Tissue

There was a slight tendency in the group of cows receiving EPS to have a higher content of C18:3 n-6 in adipose tissue than in the control group. However, energy-protein supplementation had no statistically significant effect on the content of any fatty acid in adipose tissue of cows (Figure 1 and Table S2). The percentage of saturated fatty acids, mono- and polyunsaturated (n-3 and n-6) FA in adipose tissue did not change due to EPS supplementation.

## 3. Discussion

### 3.1. The Effect of Parity and Stage of Lactation on Fatty Acid Profiles

Our results show that stage of lactation had a significant impact on several fatty acids in all examined tissues e.g., LA content in blood, liver, and adipose tissue increased, whereas ALA content in blood and CLA content in adipose tissue decreased with the progressing lactation. Parity of cows had no effect on any of the fatty acids in blood, affected only the content of C16:1 *c*9 in liver, and affected the content of most of the saturated and monounsaturated fatty acids in adipose tissue. The main animal factors that affect fatty acid profile of cattle products are breed, age, stage of lactation and individual impact of an animal [7,8]. Among the environmental factors, the most significant influences are diet and season [9]. For example, the higher levels of C16:0 and C18:1 *c*9 in blood in early stage of lactation observed in this study may be associated with increased mobilization of fatty acids from adipose tissue occurring in high-yielding dairy cows [10].

Dry matter intake was monitored daily for each cow, which allowed us to add the EPS to the total mixed ration (TMR) at a fixed proportion within the assumed next day intake. Consumption of the EPS was constant during the experiment, therefore the observed effects for stage of lactation were caused by physiological changes during lactation, and not by varying fatty acid intake from the EPS.

### 3.2. The Effect of Energy-Protein Supplementation on Fatty Acid Profiles

Due to the complex and unique composition of the energy-protein supplement used in the study, we compared our results with those of other authors based on three main components of the supplement—whole flaxseeds, protected rapeseed cake and calcium soaps of linseed and fish oils.

#### 3.2.1. Blood

The major biochemical pathway in the biohydrogenation of C18:2 n-6 into C18:1 *t*11 in the rumen leads to the formation of specific intermediates such as C18:2 *c*9, *t*11 [11]. The first step of this process is the isomerisation reaction, which converts the *c*12 double bond in the unsaturated FA to the *t*11 isomer, resulting in the formation of the conjugated diene. After the formation of *trans* bond in position 11, the *c*9 bond is hydrogenated by the action of microbiological reductases. As a result of this reaction, C18:1 *t*11 is formed. The final stage of hydrogenation of C18:2 n-6 leads to the formation of C18:0 [11]. Two groups of bacteria are involved in the hydrogenation process: bacteria responsible for the isomerisation and first hydrogenation reaction of C18:2 n-6 (e.g., *Butyrivibrio fibrisolvens*), and bacteria that perform the hydrogenation reaction of C18:1 *t*11 to C18:0 (e.g., *Fusocillus* spp.) [12]. Therefore, higher content of C18:1 *t*11 and C18:2 *c*9, *t*11 in combination with lower amount of C18:2 n-6 in blood plasma in the current study may be a result of a less complete hydrogenation of C18:2 n-6 in the rumen. The lack of effect of the EPS supplementation on the level of C18:0 in blood gives weight to this hypothesis.

Some authors found a lower concentration of C16:0 in the blood of cows during intravenous infusion of emulsion of linseed and fish oil compared to infusion of tallow [13]. Tallow has a high content of C16:0, thus an increase of this FA in blood during the supplementation is not surprising. In our study, decrease in the relative content of C16:0 in the TMR + EPS group of cows compared to control seems to have been derived by its low abundance in the EPS supplement.

In the case of negative energy balance, the mobilization of fat from adipose tissue helps to increase the concentration of C18:1 *c*9 in blood plasma. However, the cows in our study were in positive energy balance, which was documented by the results of basic milk composition. The content of urea in milk was in the range of 150–300 mg/L together with protein content of 3.2–3.6% [14], which are considered as optimal values for cows. Therefore, it can be assumed that in our study the nutrition itself was responsible for the increase of C18:1 *c*9 in blood.

As expected, the addition of the EPS caused an increase in the content of ALA in blood. EPS contains whole flaxseed and calcium soaps of linseed oil, which are characterized by high levels of ALA. Additional protection of the compounds of the EPS, which contain a high amount of ALA (no damage to the seed coat and saponification of linseed oil, as presented by Kowalski [15]) prevents intensive biohydrogenation of this FA in the rumen so that more ALA can be absorbed into blood. Increased content of LA and ALA in tissues can only be possible if the quantities of long chain fatty acids reaching the small intestine are greater than these observed in conventional feeding systems [16]. Therefore, protection of PUFA from biohydrogenation in the rumen changes the content of long chain FA in blood [17]. Physical characteristics of seeds may affect their protection against total fragmentation in mouth and may increase the rate of passage from the rumen.

The increase in the content of ALA in blood in our study was lower than in case of intravenous infusion of emulsion of linseed oil [13]. This is explained by the fact that administration of unsaturated fatty acids into the blood stream bypasses the rumen and completely prevents their hydrogenation. The increase of EPA in blood during intravenous infusion of fish oil emulsion was higher than in our experiment, also because of bypassing the rumen [13]. In our study, the EPS did not affect the content of DPA in blood, similar to case of intraruminal infusions of calcium salts of fish oil and pure fish oil [18].

#### 3.2.2. Liver

Long-chain FA in liver originate mainly from plasma non-esterified fatty acids [19]. Similar to what was observed for blood, there was no effect of the TMR + EPS diet on the content of C16:0 and C18:0 in liver. Other changes in the contents of FA in blood and liver were also similar (e.g., increased content of C18:1 *t*11, C18:1 *c*9, C18:3 n-3, C18:2 *c*9, *t*11, C20:3 n-3, C20:4 n-6, C22:1, and C20:5 n-3). Two fatty acids whose values in blood fell below the detection limit were present in the liver: C18:2 *t*10, *c*12 and C22:6 n-3. Moreover, their content increased in the liver of cows feeding on a diet supplemented with EPS.

C18:2 *t*10, *c*12 is formed by a shift in the biohydrogenation process, which occurs when the rations causing milk fat depression are fed. Instead of the isomerisation of *c*12 double bond to the *t*11 isomer (described above in Section 3.2.1), formation of the *trans* bond is observed in position 10 and C18:2 *t*10, *c*12 is formed. This FA may be further hydrogenated to C18:1 *t*10 and then to C18:0 [11]. This shift happens when rations containing a high amount of easily fermentable carbohydrates or added vegetable or fish oils are supplied as food to ruminants [20]. Because there was no decrease in milk fat in the current study [14], isomerisation of *c*12 double bond and further hydrogenation to C18:0 cannot be used to justify the absence of C18:2 *t*10, *c*12 in the blood. The reason for this observation is not clear.

We found that EPS supplementation had no effect on the content of C16:0, C16:1 *c*9, and C18:0, but increased the proportion of C18:3 n-3, which was caused by a high content of C18:3 n-3 in the flaxseed. Similar effects were observed during linseed oil infusion [13]. The increase of ALA in the TMR + EPS group was over 39% compared to the control group. The increased content of ALA in the flaxseed treated group (1.89%) compared to the control group (1.41%) was noted previously [21]. Even though slightly higher contents of ALA in liver were found in the study by Petit et al. [21], the increase in the content of this FA between the groups was lower than in the current study—34%.

Energy-protein supplement increased the proportion of n-3 fatty acids derived from fish oils—EPA, DPA and DHA in liver. Other authors did not detect EPA and DHA in liver triglycerides in cows receiving infusions of linseed oil or tallow [13]. In the case of fish oil infusions, the content of EPA and DHA was 4.69% and 5.01%, respectively. Although lower values of EPA and DHA were observed in the current experiment, it should be emphasized that fatty acid profiles of liver phospholipids and triglycerides vary considerably [17,22]. Fatty acid profile of liver in our experiment was determined without separating into fractions.

We found an increased content of n-3 fatty acids derived from fish oils, but among the n-6 fatty acids, only the content of C20:4 n-6 was significantly lower in the TMR + EPS group compared to the control. Similar effects and decreased content of all n-6 fatty acids were observed in cow liver during the supplementation with fish oil [22]. Reduced proportions of n-6 fatty acids may be a result of the competition between the incorporation of n-6 and n-3 into tissues [22].

#### 3.2.3. Adipose Tissue

Medium-chain FA (C8:0 to C12:0) in adipose tissue were in quantities below detection level. These FA are not stored in adipose tissue in significant amount, since their chains are extended to C14:0 and other long chain FA. Small amount of these FA was only observed in fat of adipose tissue in cows supplemented with coconut oil [23].

In our study there was no effect of the EPS on the content of fatty acids of adipose tissue, which agrees with previous results showing that the ration had no or little effect on the FA profile of adipose tissue [24]. According to Lake et al. [24], nutrition had little effect on FA profile of adipose tissue of cows in the early lactation, as only a small amount of FA from the ration had a chance to reach the adipocyte for storage. This was because of the high demand for nutrients during the early lactation period. In the late lactation some cows did not achieve positive energy balance, which caused the lack of effect of nutrition on the FA profile of adipose tissue in this stage of lactation [24]. In general, it was proved that long-chain fatty acids of subcutaneous fat are more resistant to modifications than FAs of mesenteric or perirenal fat [25]. In contrast, some studies showed that it was possible to modify the content of long-chain fatty acids in adipose tissue of cows during the dry period over a relatively short period of time (46 days) by changing their nutrition [17].

## 4. Materials and Methods

### 4.1. Animals, Treatments and Experimental Design

The experiment was conducted on 24 Polish Holstein-Friesian cows in full lactation (starting the experiment at 100–200 days after calving), divided into two feeding groups. Cows in the feeding groups were equally represented by the number of lactation (12 primiparous and 12 multiparous) and stage of lactation (8 cows in each of 3 groups: early, mid- and late lactation), as well as milk yield. One group of 12 cows was fed solely a total mixed ration (TMR or control), while the other group of 12 cows was fed a ration with the addition of two kg of energy-protein supplement per cow/day (TMR + EPS group). The study was performed in a cross over design with three weeks of adaptation period followed by four weeks of experimental period. Diets were then changed over—the group of cows fed the control ration during these first 7 weeks of the experiment started receiving TMR + EPS, and the group fed the TMR + EPS started receiving the control ration. The adaptation (3w) and experimental periods (4w) repeated. The total time of the experiment was 14 weeks.

The cows were healthy throughout the whole experimental period. Statistical analysis was performed to assert that mean body weight and mean body condition scores were not different between the groups (average weight and body condition score for the control group was 654 ± 72 kg and BCS 3.1 ± 0.5 respectively, whereas for the TMR + EPS group these values were 634 ± 75 kg and BCS 3.0 ± 0.4). The cows were kept in a loose housing barn. The feeding alley was equipped with 24 feeders and four water troughs. The animals had a free access to feed and water all day. The cows were milked twice a day at 5:00–6:00 a.m. and 5:00–6:00 p.m.

### 4.2. Diets and Feeding Additive

Two rations were prepared and balanced for a yield of 35 kg of milk/day (Table 1, Table 2 and Table 3)—the control TMR and the TMR supplemented with the EPS (TMR + EPS). Throughout the paper, TMR + EPS refers both to the diet with the energy-protein supplementation and to the group of cows fed the TMR + EPS diet.

Dry matter intake for individual animal was being monitored daily using Roughage Intake Control system (Insentec B.V, Marknesse, The Netherlands). This let us assume a similar next-day intake, prepare a ration for each cow allowing for 15% leftovers, and add the EPS to the TMR at a fixed proportion of 2 kg/cow per day. If the amount of leftovers was lower than 10% or higher than 20%, the amount of feed in the next day ration was corrected and the EPS was added to that corrected amount. The supplement consisted of whole golden flaxseed (30.3%), protected rapeseed cake (28.0%), calcium soaps of flaxseed and fish oils (23.8%), wheat bran (15.8%), Blattin Lacto-Fett (1.7%) and mineral-vitamin additive (0.4%), and its composition is protected by the patent (number PL405508-A1). Chemical composition of the EPS is presented in Table 2. The supplement was characterized by a beneficial content of PUFA (Table 3).

### 4.3. Tissue Sampling

All procedures were conducted under protocols approved by the III Local Ethics Committee for Experiments on Animals in Warsaw (Permission No. 68/2012).

Puncture biopsy was performed under local anesthesia to obtain approximately 2g of liver tissue (three times from each cow throughout the experiment—at 0, 7 and 14 weeks). Approximately 2 g of subcutaneous adipose tissue were excised from the tail head region under local anesthesia (three times from each cow, same as the liver biopsy). After rinsing with 0.90% *w*/*v* of NaCl, the samples were frozen in liquid nitrogen and stored at −80 °C until later analyses. Both procedures were performed according to Douglas et al. [17].

Blood samples (9 mL) were always collected 2 h after the morning feeding from the jugular vein to S-Monovette (Sarstedt, Nümbrecht, Germany) tubes with EDTA (five times from each cow throughout the experiment—at 0, 4, 7, 11, and 14 weeks). Plasma was obtained via centrifugation at 3000× *g* for 15 min at 4 °C, and then frozen at −20°C.

### 4.4. Lipid Analysis

Lipids were extracted from liver and adipose tissue using a chloroform–methanol mixture (2:1) according to Folch et al. [26]. Extraction of lipids from plasma was performed using a hexane–isopropanol mixture (3:2) [27].

Derivatization of FA into FA-methyl esters (FAMEs) was performed by transesterification method according to ISO 5509:2000 [28]. Fatty acid profiles were determined using Agilent 7890A gas chromatograph equipped with a flame ionization detector, ChemStation data processing system and Varian Select FAME column (length 100 m, internal diameter 0.25 mm, phase film thickness 0.25 μm), split 25:1. Separation was performed at pre-programmed temperatures: 120 °C for 1 min; 120–175 °C at 7.5 °C/min; 175–215 °C at 2.75 °C/min; 215–230 °C at 20 °C/min. Helium was used as the carrier gas, the pressure was constant, injector temperature was 220 °C, and detector temperature was 300 °C. The velocity of the carrier gas was equal to 23.375 cm/s. Identification of fatty acids was made on the basis of the relative retention time to palmitic acid. Quantitative analysis was carried out by external calibration for all fatty acids from Sigma-Aldrich (now Merck KGaA, Darmstadt, Germany).

### 4.5. Statistical Analysis

Fatty acid data were tested for normality using the Shapiro–Wilk test. FA data sets that did not have a normal distribution were subjected to logarithmic transformation. Due to the varied FA composition of tissues, the analysis included 16 fatty acids in the blood, 22 fatty acids in the liver and 20 fatty acids in the adipose tissue.

We divided all the cows in two parity groups and three stage of lactation groups to help compare the results with those of other authors, as such groups were the most common in the literature [7]. The cows were divided into two groups in terms of parity (primiparous and multiparous), and three groups according to their stage of lactation: early (up to 120 days in milk), middle (120–200 days in milk) and late (over 200 days in milk). Because the experiment lasted 14 weeks, cows which started the experiment around 200th day of lactation were in their late stage of lactation at the end of the experiment.

The following single-trait repeatability model fitting fixed supplementation, parity, and stage of lactation effects as well as random phenotypic cow effect was applied to analyze the FA variances:

y_ijklm_ = µ + EPS_i_ + P_j_ + S_k_ + a_l_ + e_ijklm_ (1)

where y_ijklm_ is the content of the FA in tissue (blood plasma, liver or adipose tissue), µ is the mean content of the FA in tissue, EPS_i_ is the fixed effect of the energy-protein supplement, P_j_ is the fixed effect of parity, S_k_ is the fixed effect of stage of lactation, a_l_ is the random phenotypic effect of animal and e_ijkim_ is the random residual effect. Preliminary data analysis showed that there were no interactions between any of the main effects (diet, parity and stage of lactation), therefore only these three main factors were included in the model.

Bonferroni correction was used in the post-hoc comparisons between the groups.

## 5. Conclusions

In this paper, we examined the effect of the stage of lactation, parity and specially designed energy-protein supplement on the content of fatty acids in three different tissues—blood plasma, liver and adipose tissue. We found multiple relationships between lactation and parity and FA composition of tissues: stage of lactation had a significant impact on several fatty acids in all examined tissues; e.g., LA content in blood, liver, and adipose tissue increased, whereas ALA content in blood and CLA content in adipose tissue decreased with the progressing lactation. Parity of cows had no effect on any of the fatty acids in blood, affected only the content of C16:1 *c*9 in liver, and affected the content of most of the saturated and monounsaturated fatty acids in adipose tissue. Diet supplementation affected the content of the similar fatty acids in blood and liver (e.g., increased the content of C18:3 n-3, C18:2 *c*9, *t*11, and C20:5 n-3), but had no effect on the content of any FA in the adipose tissue. Supplementation with the EPS led to increased concentration of PUFA in blood and their availability for other tissues, showed by a subsequent increase of PUFA in liver. It is well-known that PUFA act as mediators in a series of processes in several reproductive tissues. Therefore the observed increase of PUFA may have a positive effect on the reproductive performance of cattle, such as higher pregnancy rate, greater number of follicles, or a faster restart of ovulation postpartum (reviewed by Herrera-Camacho [6]). These results will also help to better follow and understand metabolic pathways of fatty acids not only in relation to modified nutrition, but also to parity and stage of lactation. Understanding a destination of different fatty acids in cow’s organism in respect to these factors may in turn contribute to enhancing the positive impact of fat supplementation (such as altering the reproductive performance), and may help farmers to choose the appropriate timing of supplementation (such as cow’s age and stage of lactation).

## Figures and Tables

**Table 1:** The composition of the total mixed ration (TMR).

Component	Dry Matter
Forage:Concentrates (%)	67:33
Corn silage (kg)	9.4
Grass silage (kg)	4.0
Wheat straw (kg)	1.8
Silaged grains of corn (kg)	0.7
Dried molassed beet pulp (kg)	1.8
Concentrates ^a^ (kg)	5.4

^a^ Composition of concentrates (in % on a dry matter basis): triticale, 38.14; soybean meal, 52.00; fodder chalk, 2.42; CO-BIND^®^ A-Z (Delacon, Stara Iwiczna, Poland), 0.33; sodium bicarbonate, 3.23; Vitamix KW (Polmass, Bydgoszcz, Poland), 2.42; NaCl, 0.65; MgO, 0.81.

**Table 2:** Chemical composition (g/kg of dry matter) of the used mixed feeds: mixed ration or control (TMR), energy-protein supplement (EPS) and supplemented mixed ration (TMR + EPS).

Chemical Composition	TMR	TMR + EPS	Energy-Protein Supplement (EPS)
Crude ash	92	89	79
Protein	140	144	188
Crude fat	27	48	319
Crude fibre	211	202	105
Neutral detergent fibre	430	412	271
Starch	161	150	12

**Table 3:** Fatty acid composition (g/100g of total fatty acids) of the used mixed feeds: mixed ration or control (TMR), energy-protein supplement (EPS), and supplemented mixed ration (TMR + EPS).

Fatty Acid (g/100 g)	TMR	TMR + EPS	Energy-Protein Supplement (EPS)
C12:0	0.28	0.16	0.12
C14:0	0.62	0.87	1.32
C14:1 *c*9	0.02	0.02	0.03
C15:0	0.08	0.07	0.11
C16:0	16.75	14.28	10.39
C16:1 *c*9	0.73	0.96	1.59
C18:0	2.54	2.97	3.99
C18:1 *c*9	21.95	28.63	42.61
C18:2 n-6	38.32	28.44	15.07
C18:3 n-3	15.53	21.12	21.00
C20:1	0.14	0.34	1.33
C20:2 n-6	0.05	0.13	0.60
C20:3 n-6	0.05	0.21	0.28
C20:3 n-3	0.05	0.09	0.20
C20:4 n-6	0.00	0.04	0.04
C20:5 n-3	0.03	0.12	0.12

## Supplementary Materials

The following are available online. Tables S1–S2.
