# Supplementary material for: Energy-Protein Supplementation and Lactation Affect Fatty Acid Profile of Liver and Adipose Tissue of Dairy Cows
Source: Molecules. 2018 Mar 9;23(3):618. doi: 10.3390/molecules23030618 (PMC6017836; doi:10.3390/molecules23030618)
Supplement: Supplementary file 1 [file molecules-23-00618-s001.pdf]

Supplementary Materials

Energy-Protein Supplementation and Lactation Affect Fatty Acid Profile of Liver and Adipose Tissue of Dairy Cows

Anna M. Brzozowska, Marek Lukaszewicz and Jolanta M. Oprzadek*

Institute of Genetics and Animal Breeding of the Polish Academy of Sciences, Postepu str. 36A, 05-552 Jastrzebiec, Poland; aa.brzoz@gmail.com (A.M.B.); m.lukaszewicz@ighz.pl (M.L.)

* Correspondence: j.oprzadek@ighz.pl; Tel.: +48-227367118; Fax: +48-227561417

Table S1. Fatty acids included in statistical analyses in the study.

Lipid Numbers	Systematic Name	Common/Other Name
C10:0	Decanoic acid	Capric acid
C14:0	Tetradecanoic acid	Myristic acid
C14:1 *c*9	(Z)-9-Tetradecenoic acid	Myristoleic acid
C15:0	Pentadecanoic acid	Pentadecylic acid
C16:0	Hexadecanoic acid	Palmitic acid
C16:1 *c*9	(Z)-9-Hexadecenoic acid	Palmitoleic acid
C18:0	Octadecanoic acid	Stearic acid
C18:1 *t*11	(E)-11-Octadecenoic acid	Vaccenic acid
C18:1 *c*9	(Z)-9-Octadecenoic acid	Oleic acid
C18:2 n-6	(Z,Z)-9,12-Octadecadienoic acid	Linoleic acid
C18:3 n-6	(Z,Z,Z)-6,9,12-Octadecatrienoic acid	Gamma-linolenic acid
C18:3 n-3	(Z,Z,Z)-9,12,15-Octadecatrienoic acid	Alpha-linolenic acid
C18:2 *t*10, *c*12 (CLA[1])	(E, Z)-10,12-Octadecadienoic acid	
C18:2 *c*9, *t*11 (CLA[1])	(Z,E)-9,11-Octadecadienoic acid	Rumenic acid
C20:0	Eicosanoic acid	Arachidic acid
C20:1	(Z)-11-Eicosenoic acid	Gondoic acid
C20:3 n-3	(Z,Z,Z)-11,14,17-Eicosatrienoic acid	
C20:4 n-6	(Z,Z,Z,Z)-5,8,11,14-Eicosatetraenoic acid	Arachidonic acid
C20:3 n-6	(Z,Z,Z)-8,11,14-Eicosatrienoic acid	Dihomo-gamma-linolenic acid
C22:1	(Z)-13-Docosenoic acid	Erucic acid
C20:5 n-3	(Z,Z,Z,Z,Z)-5,8,11,14,17-icosapentaenoic acid	Timnodonic/Eicosapentaenoic acid
C22:5 n-3	(Z,Z,Z,Z,Z)-7,10,13,16,19-docosapentaenoic acid	Docosapentaenoic acid
C22:6 n-3	(Z,Z,Z,Z,Z,Z)-4,7,10,13,16,19-docosahexaenoic acid	Docosahexaenoic acid

[1]CLA - conjugated linoleic acids

Table S2. Least squares means (LSM) and standard errors (SE) of individual fatty acids in blood, liver and adipose tissue in the control (total mixed ration) and TMR + EPS (ration plus the energy-protein supplement) groups of cows (g/100g of total fatty acids).

	Blood		Liver		Adipose tissue	
Fatty acid (g/100g)	Control group LSM (SE)	TMR + EPS group LSM (SE)	Control group LSM (SE)	TMR + EPS group LSM (SE)	Control group LSM (SE)	TMR + EPS group LSM (SE)
C14:0	0.30* (1.05)	0.27* (1.05)	0.58 (1.64)	0.59 (1.06)	2.85 (0.11)	2.92 (0.12)
C14:1 *c*9	NA[2] (NA)	NA (NA)	0.05 (1.17)	0.06 (1.16)	1.94 (1.07)	2.06 (1.07)
C15:0	0.56** (1.02)	0.50** (1.02)	0.39** (1.03)	0.36** (1.03)	0.43 (0.02)	0.42 (0.02)
C16:0	15.01* (0.22)	14.56* (0.23)	10.89 (1.03)	10.88 (1.03)	24.43 (0.52)	24.98 (0.55)
C16:1 *c*9	0.33 (1.08)	0.30 (1.08)	0.62 (1.10)	0.61 (1.10)	9.00 (1.06)	9.04 (1.06)
C18:0	29.22 (1.01)	29.25 (1.01)	32.16 (0.34)	31.66 (0.38)	7.60 (0.46)	7.28 (0.49)
C18:1 *t*11	0.38* (1.05)	0.42* (1.06)	0.73* (1.06)	0.85* (1.06)	1.13 (1.18)	2.32 (1.27)
C18:1 *c*9	10.53** (0.38)	11.80** (0.40)	12.64* (1.04)	13.21* (1.04)	48.78 (0.58)	48.49 (0.61)
C18:2 n-6	30.61* (0.51)	29.68* (0.54)	11.78* (0.24)	12.09* (0.25)	1.60 (1.04)	1.62 (1.04)
C18:3 n-6	0.38** (1.05)	0.29** (1.05)	0.27 (1.11)	0.23 (1.13)	0.21 (0.02)	0.24 (0.02)
C18:3 n-3	2.67** (0.14)	3.43** (0.14)	1.05** (1.06)	1.47** (1.06)	0.25 (1.06)	0.26 (1.06)
C18:2 *t*10, *c*12	NA (NA)	NA (NA)	0.06** (1.05)	0.08** (1.06)	0.05 (1.20)	0.07 (1.22)
C18:2 *c*9, *t*11	0.20* (0.01)	0.24* (0.01)	0.22** (0.01)	0.27** (0.01)	0.76 (1.07)	0.76 (1.08)
C20:0	NA (NA)	NA (NA)	0.07** (0.01)	0.12** (0.01)	0.20 (0.01)	0.20 (0.01)
C20:1	NA (NA)	NA (NA)	0.12 (1.05)	0.12 (1.05)	0.22 (1.05)	0.22 (1.05)
C20:3 n-3	4.48** (0.14)	3.59** (0.15)	8.86** (0.31)	7.00** (0.33)	0.06 (1.08)	0.05 (1.09)
C20:4 n-6	3.13** (1.03)	2.78** (1.03)	9.44** (1.02)	8.69** (1.02)	0.07 (1.14)	0.06 (1.15)
C20:3 n-6	NA (NA)	NA (NA)	0.07 (1.06)	0.07 (1.07)	NA (NA)	NA (NA)
C22:1	0.63** (0.04)	0.83** (0.05)	1.72** (0.11)	2.25** (0.12)	0.02 (1.07)	0.02 (1.07)
C20:5 n-3	0.59** (0.04)	0.77** (0.04)	1.79** (1.05)	2.37** (1.05)	NA (NA)	NA (NA)
C22:5 n-3	1.30 (1.04)	1.24 (1.04)	5.04** (0.17)	5.50** (0.19)	0.04 (1.16)	0.04 (1.18)
C22:6 n-3	NA (NA)	NA (NA)	0.49** (0.03)	0.62** (0.03)	0.02 (1.18)	0.02 (1.29)
Σn-6	34.02** (0.53)	32.68** (0.56)	21.64** (0.27)	21.14** (0.28)	1.89 (1.04)	1.93 (1.04)
Σn-3	9.05 (0.17)	8.99 (0.18)	17.35 (0.33)	17.09 (0.37)	0.37 (0.02)	0.35 (0.03)
ΣSFA[1]	45.07 (1.01)	44.89 (1.01)	44.22 (0.20)	43.77 (0.23)	35.61 (0.86)	35.87 (0.90)
ΣMUFA[1]	11.41** (1.03)	13.07** (1.03)	16.08** (1.03)	17.33** (1.03)	61.28 (0.87)	61.02 (0.91)
ΣPUFA[1]	43.08* (0.57)	41.73* (0.60)	39.22* (0.43)	38.53* (0.47)	3.12 (0.11)	3.13 (0.11)

[1] ΣSFA – the sum of saturated fatty acids, ΣMUFA – the sum of monounsaturated fatty acids, ΣPUFA – the sum of polyunsaturated fatty acids; [2] NA - non applicable (fatty acid was not detectable in the sample); Statistically significant differences in the tissue fatty acid composition between the control and TMR + EPS groups of cows are indicated by * ($p<0.05$) and ** ($p<0.01$).
